# Body Image Concerns and Associated Factors up to Five Years After Cancer in Young Adulthood: A Swedish Longitudinal Population‐Based Study

**DOI:** 10.1002/pon.70545

**Published:** 2026-07-17

**Authors:** Rebecca Skog, Sarah Marklund, Claudia Lampic, Lena Wettergren

**Affiliations:** ^1^ Department of Public Health and Caring Sciences Uppsala University Uppsala Sweden; ^2^ Department of Psychology Umeå University Umeå Sweden; ^3^ Department of Women's and Children's Health Karolinska Institutet Stockholm Sweden

**Keywords:** body image, cancer, longitudinal studies, neoplasms, psychological distress, young adult

## Abstract

**Objective:**

This study aims to investigate prevalence of body image disturbance and how perceptions of the body evolve from 1.5 to 5 years following a cancer diagnosis in young adulthood. Additionally, to identify factors associated with worse body image over time.

**Methods:**

A population‐based longitudinal study with 1010 individuals diagnosed with breast‐, cervical‐, ovarian‐, testicular cancer, brain tumor or lymphoma at ages 18–39 years was conducted. Participants were approached with surveys 1.5, 3 and 5 years post‐diagnosis. Body image was assessed using the Body Image Scale (BIS). Linear mixed models with random intercepts were used to investigate change in body image.

**Results:**

Approximately half of females and a fourth of males reported clinical levels of body image disturbance (≥ 10) at 1.5 years post diagnosis, followed by ∼40% and ∼15% respectively at follow‐ups. The highest level of body image disturbance was reported among those diagnosed with breast cancer and lymphoma. Body image improved over time among male survivors and females diagnosed with breast cancer and lymphoma (*p* < 0.001), while other groups remained stable (all *p* > 0.05). Factors associated with worse body image in both models included intensive treatment and emotional distress.

**Conclusions:**

Although body image was found to improve over time among males and females with breast cancer and lymphoma, a substantial proportion report persistent body image disturbance at follow‐up. Future research should employ qualitative methods in exploring the experiences and needs of young adults following cancer, in order to develop timely support for alleviating body image problems.

## 1 Introduction

A growing population of young adults (18–39 years) are living with and beyond cancer, and they face unique challenges due to simultaneously navigating age‐related milestones [1]. Such milestones include making and fulfilling potential family plans, exploring and establishing sexual‐ and romantic relationships, and developing the basis for a positive body image [1]. Cancer and its treatment can lead to a variety of changes to appearance (e.g., scarring, hair loss), bodily sensations (e.g., pain, numbness) and bodily functions (e.g., erectile dysfunction, infertility), as well as to feelings toward and perceptions of one's body [2, 3]. Experiencing such changes during a time in life where body image is of particular importance [1, 4] can negatively impact sense of self and satisfaction with the body [3, 5] and quality of life [6], including psychological well‐being [7].

Body image is a multidimensional construct, involving affective, cognitive and behavioral dimensions [8]. In the cancer context, body image disturbance refers to self‐perceived unappreciated change in appearance and/or physical functioning or in one's perceptions of the body, and experiencing psychological distress regarding such changes [9]. Concerns about the body is common following cancer [2], however prevalence of image concerns among young adult cancer survivors varies across previous studies. In a scoping review on body image concerns among adolescents and young adults (AYAs) (13–39 years) [10], prevalence ranged from 17% to 63%. The lowest prevalence was reported among long‐term survivors of testicular cancer, and the highest among a sample of recently diagnosed young adults (a sample consisting primarily of females with mixed cancer types) [10]. These differences reflect two of the factors known to be related to body image concerns: sex and time since diagnosis. Generally, females report more body image issues compared to males [11], and impact on body image appear to be most bothersome close to diagnosis and treatment initiation and improve over time [11, 12, 13]. Other factors found to be associated with higher levels of body image concerns include various physical/clinical factors (e.g., type of cancer [11] and treatment [11], sexual dysfunction [14]), psychological factors (e.g., satisfaction with sex life [14], symptoms of anxiety and depression/psychological distress [15]), interpersonal factors (e.g., lower relationship satisfaction [16], marital status [6]) and demographic/contextual factors (e.g., educational level [17], age [17]).

Despite existing research on body image among young adults following cancer, important limitations remain. A majority of previous studies have focused solely on survivors of breast cancer, and few have explicitly investigated body image among males and among certain types of cancer, for example lymphoma and brain tumors. Further, existing studies have primarily been cross‐sectional, limiting our understanding of how body image evolves over time. Results from the limited number of longitudinal studies included in the scoping review by Vani et al. [10] show that young adults report lower satisfaction with their appearance during the first 6 months following surgery, and that negative impact on physical appearance was reported up to 2 years post‐diagnosis. However, there is limited knowledge about how long‐term body image evolves following cancer diagnosis/treatment in young adulthood.

This study aims to investigate prevalence of body image disturbance and how perceptions of the body evolve from 1.5 to 5 years following a cancer diagnosis in young adulthood. Further, Additionally, the study aims to identify factors associated with worse body image over time.

## 2 Methods

### 2.1 Study Setting and Design

This population‐based longitudinal study is part of the Fertility and Sexuality following Cancer (Fex‐Can) cohort study (see study protocol [18]). The present study body image from 1.5 up to 5 years post‐diagnosis and adheres to the Strengthening the Reporting of Observational Studies in Epidemiology (STROBE) guidelines for cohort studies [19].

### 2.2 Participants and Procedure

**FIGURE 1:**
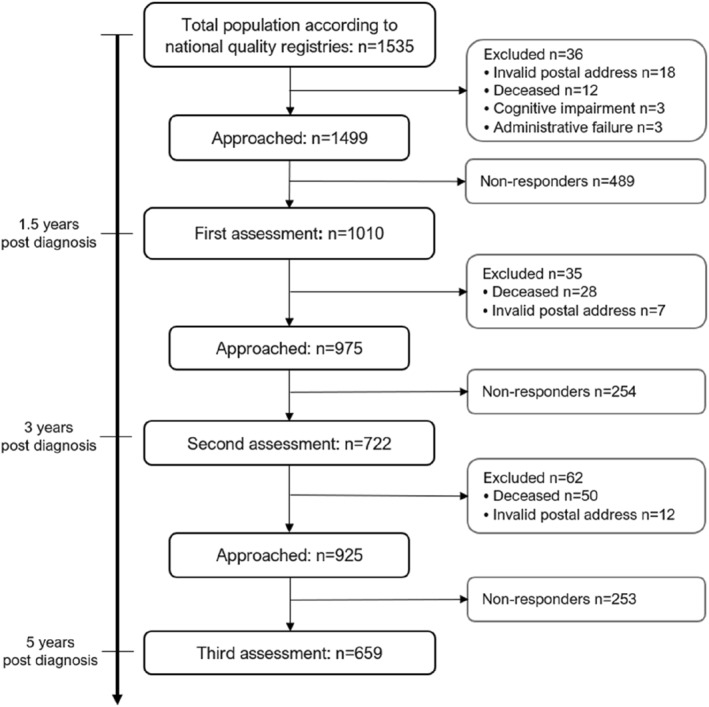
Flow diagram of participants.

All young adults aged 18–39 years when diagnosed with breast‐, cervical‐, ovarian‐, testicular cancer, lymphoma or brain tumor were identified via Swedish cancer quality registries. From a pool of 1535 eligible individuals, 36 were excluded due to invalid postal address (*n* = 18), death (*n* = 12), cognitive impairment (*n* = 3) and administrative failure (*n* = 3). Thus, a total of 1499 individuals were contacted ∼1.5 years post‐diagnosis (Figure 1). Potential participants received study information and the first survey (available online or on paper). Non‐responders received two reminders. Those declining participation or not completing the first assessment were excluded from receiving subsequent surveys. Incentives included cinema tickets for completing assessments. Ethical approval for the study procedures was obtained from the Regional Ethic Review Board in Stockholm (Registration number: 2013/1746‐31/4; 2014/2244‐32; 2015/2042‐32/4; 2016/1848‐32, 2017/916‐32, 2017/1416‐32) and was conducted in accordance with the principles of the Declaration of Helsinki. Written informed consent was obtained from all participants prior to answering the surveys.

### 2.3 Data Collection

Comprehensive surveys were completed at 1.5, 3 and 5 years following diagnosis. For the present study, the variables from the survey that were selected included several clinical‐ and sociodemographic variables, body image, and emotional distress.

### 2.4 Measures

#### 2.4.1 Primary Outcome

##### 2.4.1.1 Body Image

Body image was assessed using the Body Image Scale (BIS), which was developed as a brief scale for assessing body image in cancer patients [20]. The scale consists of 10 items covering affective (i.e., emotional responses and feelings toward the body), cognitive (i.e., thoughts, beliefs and perceptions of one's body) and behavioral items (i.e., behaviors affected by one's feelings and thoughts about the body). Examples of items include “Have you felt less physically attractive as a result of your disease or treatment?” (affective), “Have you felt dissatisfied with your body?” (cognitive) and “Did you avoid people because of the way you felt about your appearance?” (behavioral). Participants are instructed to consider their experiences within the past week when responding, and responses are reported on a four‐point Likert scale ranging from 0 = “Not at all” to 3 = “Very much”. Scores were calculated by the sum of responses ranging from 0 to 30, with higher scores indicating higher levels of body image disturbance. A total score of ≥ 10 has been suggested to reflect body image disturbance reaching a clinical level, hereafter referred to as BIS cases [21]. The BIS has demonstrated satisfactory convergent validity, internal consistency, and test‐retest reliability, and has been shown to be sensitive to detect change over time [20, 22]. Cronbach's alpha for BIS was high among both the female (*α* = 0.93) and male (*α* = 0.90) sample at 1.5 years.

#### 2.4.2 Covariates

##### 2.4.2.1 Emotional Distress

The Hospital Anxiety and Depression Scale (HADS) was used to assess emotional distress [23]. The scale consists of 14 items, with a 7‐item anxiety and a 7‐item depression subscale. Responses are recorded on a 4‐point Likert scale (0–3), and scores are combined into an overall score representing emotional distress (range 0–42). Higher HADS scores indicating greater emotional distress. The scale has demonstrated convergent and discriminant validity, internal consistency, and test‐retest reliability in cancer populations [23, 24].

##### 2.4.2.2 Sociodemographic and Clinical Data

Data on sex, age at diagnosis, cancer type and treatment received were collected from the Swedish national quality registries. To enable comparison of treatments across diagnoses, intensity of treatment was classified according to the adapted version of the Intensity of Treatment Rating Scale (ITR 3.0): the ITR‐Young Adult (YA) [25]. Each participant is thus categorized into one of four intensity levels (least/moderately/very/most intensive/extensive) based on diagnosis, stage and the treatment modalities. Time‐sensitive variables were collected at all assessments, including highest completed or ongoing level of education, occupational status, current partner status, and sexual orientation. Participants also provided self‐report data on ongoing cancer treatment at all assessments. At 1.5 years, participants further reported their country of birth.

##### 2.4.2.3 Statistical Analysis

Attrition analyses were conducted using Chi‐squared tests for categorical variables and two‐tailed independent *t*‐tests or Mann‐Whitney U‐tests for continuous variables, depending on distribution of data. All tests were two‐tailed and *p*‐values < 0.05 were considered statistically significant. To investigate how body image evolves over time, Linear Mixed Models (LMMs) [26] were fitted using the R packages Ime4 and ImerTest. Given prior evidence of sex differences in body image [11], models were fitted separately by sex. A hierarchical model‐building approach was used, starting with simple models with fixed effects for time and random intercepts to account for baseline differences in body image and within‐subject correlation. Candidate fixed effects and interaction terms were added step‐by‐step and compared using likelihood ratio tests and Akaike's Information Criterion (AIC), with the goal of identifying a parsimonious model.

Candidate covariates included both time‐variant and time‐invariant variables. Time‐variant variables included sexual orientation (other/heterosexual), relationship status (not partnered/partnered), highest completed or ongoing level of education (not university/university), occupational status (not working or studying/working or studying), ongoing cancer treatment (yes/no), and emotional distress (continuous). Time‐invariant variables included age at diagnosis (continuous), country of birth (not Sweden/Sweden), age at study entry (continuous), diagnosis (breast/cervical/ovarian/brain tumor/lymphoma/testicular) and treatment intensity (least‐moderately/very‐most). Variables that improved model fit were retained in the final models.

Variables associated with non‐response at follow‐ups were included in models to adjust for potential bias. Residual diagnostics of the models indicated some non‐random patterns. To account for potential heteroscedasticity and non‐normality, both model‐based and robust standard errors were compared, which yielded similar results. Post hoc analyses were performed using estimated marginal means (EMMs), assessed using the emmeans package in R. Pairwise comparisons were conducted with Tukey‐adjusted *p*‐values to control for multiple comparisons. All statistical analyses were performed in R version 4.2.2 [27].

## 3 Results

### 3.1 Sample Characteristics

A total of 1010 participants responded to the survey at 1.5 years, followed by 722 and 659 at 3 and 5 years, respectively. Of those who completed the survey at 1.5 years, 791 (78%) also completed either or both of the follow‐ups.

Sociodemographic and clinical characteristics of study participants are presented in Table 1. At all assessments, a majority of participants were born in Sweden, partnered, and currently working or studying. Mean age at diagnosis was 33 years (SD 4.9) among females and 31 years (SD 5.5) among males. Approximately 50% of female and 40% of male participants had received treatment classified as either very or most intensive/extensive. Breast‐ and testicular cancer were the most common cancer types for females and males, respectively.

**TABLE 1:** Sociodemographic and clinical characteristics of participants at 1.5 years post‐diagnosis (*n* = 1010).

	Females	Males
	*n* = 694	*n* = 316
	*n* (%)	*n* (%)
Sociodemographic variables		
Age at diagnosis, years		
Mean (SD)	33.2 (4.9)	30.7 (5.5)
Country of birth		
Sweden	579 (83.5)	272 (86.3)
Outside Sweden	114 (16.5)	43 (13.7)
Missing	1	1
Sexual orientation		
Heterosexual	633 (93.4)	297 (95.5)
Other^a^	45 (6.6)	14 (4.5)
Missing	16	5
Relationship status		
Partnered	585 (84.7)	245 (77.5)
Not partnered	106 (15.3)	71 (22.5)
Missing	3	—
Educational level		
University	417 (60.3)	142 (44.9)
Not university^b^	275 (39.7)	174 (55.1)
Missing	2	—
Occupational status		
Working or studying	530 (76.6)	269 (85.1)
Not working/studying^c^	162 (23.4)	47 (14.9)
Missing	2	—
Clinical variables		
Diagnosis		
Breast cancer	349 (50.3)	—
Cervical cancer	190 (27.4)	—
Ovarian cancer	32 (4.6)	—
Brain tumor	66 (9.5)	57 (18.0)
Lymphoma	57 (8.2)	59 (18.7)
Testicular cancer	—	200 (63.3)
Treatment intensity^d^ ^,^ ^e^		
Very/most intensive/extensive	359 (53.3)	126 (40.4)
Least/moderately intensive/extensive	314 (46.7)	186 (59.6)
Missing	21	4
Ongoing cancer treatment		
Yes^e^ ^,^ ^f^	250 (36.3)	38 (12.1)
No	438 (63.7)	276 (87.9)
Missing	6	2
Psychological variables		
Emotional distress		
Mean (SD)	13.7 (7.4)	10.5 (6.9)
Missing	8	3

*Note:* Numbers do not sum up due to missing data.

^a^ Includes homosexual, bisexual, other.

^b^ Includes elementary school, upper secondary school, folk high school.

^c^ Includes unemployed, sick‐leave, retired, parental leave, other.

^d^ Classified according to the ITR‐YA [25].

^e^ Additional treatment factors by diagnosis are presented in Table S6.

^f^ Includes chemotherapy, radiotherapy, endocrine therapy, other.

Comparison between responders and non‐responders at the 1.5‐year assessment have been reported previously [28]. In summary, male non‐responders were younger at time of diagnosis compared to responders, and response rates among females differed by cancer type, with lower participation among females diagnosed with ovarian cancer. Additional attrition analyses were conducted comparing 1.5‐year clinical and sociodemographic characteristics between those who responded only at 1.5 years and those who completed one or two more additional surveys. Females who responded only to the 1.5‐year assessment were more likely to be born outside Sweden (*p* = 0.002), not have university level education (*p* < 0.001), and have higher emotional distress (*p* = 0.015) and body image scores (*p* = 0.014). Among males, those who responded only to the 1.5‐year assessment were more likely to be born outside Sweden (*p* = 0.022) and not currently working or studying (*p* = 0.006). No differences were found among females and males for treatment intensity, relationship status and other clinical‐ and sociodemographic variables (Table S1).

### 3.2 Body Image Summary Scores at 1.5, 3 and 5 Years Post‐Diagnosis

Body Image Scale scores and proportion of participants reporting a BIS score of ≥ 10 are shown by sex and type of cancer in Table 2. Overall, BIS scores were highest at 1.5 years, with approximately half of female (54.0%) and a fourth (23.8%) of male participants reaching the cut‐off for clinically relevant body image disturbance.

**TABLE 2:** Body image summary scores and prevalence of BIS cases (BIS score > 10) by diagnosis.

	BIS score/cases 1.5 years		BIS score/cases 3 years		BIS score/cases 5 years	
	Mean (SD)	*n* (%)	Mean (SD)	*n* (%)	Mean (SD)	*n* (%)
Total						
Females	11.0 (8.2)	374 (54.0)	9.9 (7.4)	219 (43.8)	9.4 (7.1)	189 (40.8)
Males	6.1 (6.0)	75 (23.8)	4.8 (5.2)	33 (15.3)	4.7 (5.3)	30 (15.3)
Breast cancer	14.0 (8.1)	225 (64.7)	11.5 (7.6)	135 (52.1)	10.1 (7.0)	105 (43.6)
Cervical cancer	8.4 (6.8)	67 (35.4)	7.2 (6.3)	38 (29.9)	8.1 (6.6)	42 (35.5)
Ovarian cancer	9.7 (7.4)	12 (37.5)	9.3 (6.4)	9 (39.1)	10.0 (7.5)	10 (45.5)
Brain tumor						
Females	9.2 (7.6)	26 (39.4)	8.0 (6.6)	18 (33.3)	8.3 (7.6)	16 (34.8)
Males	6.2 (5.8)	17 (29.8)	4.9 (5.6)	8 (21.1)	5.1 (5.4)	6 (16.2)
Lymphoma						
Females	15.5 (8.4)	44 (77.2)	11.2 (8.2)	19 (51.4)	10.5 (7.3)	16 (45.7)
Males	8.6 (7.0)	23 (39.0)	6.0 (5.7)	5 (13.2)	6.0 (6.0)	7 (20.6)
Testicular cancer	5.3 (5.5)	35 (17.6)	4.4 (4.9)	20 (14.3)	4.2 (5.0)	17 (13.6)

### 3.3 Change in Body Image Over Time Among Females

The final fitted model for females included a random intercept capturing individual variability and time as a fixed effect. Level of treatment intensity and emotional distress were included as covariates. An interaction term between time and type of cancer improved the model fit and was thus retained. The final model explained 36.6% of the variance in body image through fixed effects alone, and 74.5% when including both fixed and random effects (Table S2).

There was a main effect of time (*F* = 13.43, *p* < 0.001), indicating that body image changed across the study period. Compared to 1.5‐ years post diagnosis, body image scores were significantly lower at 3 and 5 years (Table 3).

**TABLE 3:** Summary of fixed effects estimates from linear mixed models, females (*n* = 663^a^).

Fixed effect	Estimate (β)	SE	95% CI	*t*	*p*
Time					
Intercept^b^	13.52	0.82	11.91–15.13	16.52	**< 0.001**
3 years	−1.58	0.36	−2.28 to −0.88	−4.43	**< 0.001**
5 years	−2.73	0.37	−3.46 to −1.99	−7.31	**< 0.001**
Diagnosis					
Breast cancer (ref)					
Cervical cancer	−5.02	0.59	−6.18 to −3.86	−8.51	**< 0.001**
Ovarian cancer	−4.56	1.23	−7.05 to −2.07	−3.70	**< 0.001**
Brain tumor	−4.22	0.90	−6.00 to −2.45	−4.71	**< 0.001**
Lymphoma	0.99	0.99	−0.99 to 2.97	1.00	0.320
Emotional distress	0.27	0.03	0.21–0.33	8.65	**< 0.001**
Intensity of treatment					
Least/moderately (ref)					
Very/most	1.57	0.48	0.63–2.51	3.29	**0.001**

*Note:* The model is adjusted for birth country, level of education at 1.5 years, occupational status at 1.5 years, and HADS score at 1.5 years. The full fixed and random effects table is located in Table S2. Bolded values represent statistical significant *p*‐values.

^a^ 31 participants were excluded due to missing either all outcome data or covariates.

^b^ The intercept represents the expected BIS score at the reference level/zero of all variables.

However, an interaction effect between time and type of cancer was found (*F* = 4.35, *p* < 0.001), meaning that the effect of time on body image differed by cancer type. Estimated marginal means and pairwise comparisons were performed to explore the interaction within each diagnostic group at each time point (Table S3, Figure S1). BIS scores decreased significantly across the entire study period among females diagnosed with breast cancer (mean difference 1.5–5 years = 2.73 [95% CI: 2.04–3.41], *p* < 0.001) and lymphoma (mean difference 1.5–5 years = 4.38 [95% CI: 2.64–6.13], *p* < 0.001). Among breast cancer survivors, body image further improved from 1.5 to 3 years (*p* < 0.001), and between 3 and 5 years (*p* = 0.004). While BIS scores decreased between 1.5 and 3 years among participants with lymphoma (*p* < 0.001), no significant change was observed between the later assessments (*p* = 0.850). Body image among females diagnosed with cervical cancer, ovarian cancer and brain tumors did not change significantly across the study period (all *p* > 0.05).

### 3.4 Change in Body Image Over Time Among Males

The final fitted models for males included time as a fixed effect and a random intercept to account for individual‐level variability, and type of cancer, treatment intensity, ongoing treatment, and emotional distress included as covariates. The final model explained 27.4% of the variance in body image through fixed effects alone, and 72.7% when including both fixed and random effects (Table S4).

There was a significant main effect of time (*F* = 8.57, *p* < 0.001), indicating that body image scores changed across the study period. Compared to 1.5 years post diagnosis, BIS scores were significantly lower at 3‐years and 5‐years, adjusting for other covariates included in the model (Table 4).

**TABLE 4:** Summary of fixed effects estimates from linear mixed models, males (*n* = 308^a^).

Fixed effect	Estimate (*β*)	SE	95% CI	*t*	*p*
Time					
Intercept^b^	4.60	0.82	2.96–6.24	5.61	**< 0.001**
3 years	−0.87	0.27	−1.39 to −0.34	−3.27	**0.001**
5 years	−1.06	0.30	−1.65 to −0.47	−3.55	**< 0.001**
Diagnosis					
Testicular cancer (ref)					
Brain tumor	0.74	0.67	−0.58 to 2.07	1.12	0.268
Lymphoma	0.97	0.80	−0.62 to 2.56	1.21	0.229
Emotional distress	0.36	0.03	0.29–0.43	10.34	**< 0.001**
Intensity of treatment					
Least/moderately (ref)					
Very/most	1.44	0.58	0.29–2.58	2.48	**0.014**
Ongoing cancer treatment					
No (ref)					
Yes	1.15	0.75	−0.36 to 2.66	1.54	0.131

*Note:* The model is adjusted for birth country, age at diagnosis, and occupational status at 1.5 years. The full fixed and random effects table is located in Table S4. Bolded values represent statistical significant *p*‐values.

^a^ 8 participants were excluded due to missing either all outcome data or covariates.

^b^ The intercept represents the expected BIS score at the reference level/zero of all variables.

Estimated marginal means and pairwise comparisons were performed to further explore the main effect of time on body image (Table S5). While BIS scores decreased across the full study period and between the earlier assessments, no change was observed between the later assessments (*p* = 0.789), indicating that improvements in body image between 1.5 and 3 years were maintained over time.

### 3.5 Factors Associated With Worse Body Image Over Time

Several covariates were found to be associated with body image across the study period among both females and males (Tables 3 and 4, Tables [Link], [Link] and S4). In both models, participants who had received treatment classified at very/most intensive/extensive reported significantly higher BIS scores, as compared to those who received less intensive/extensive treatment (females: *β* 1.57, 95% CI [0.63–2.51], *p* = 0.001, males: *β* 1.44, 95% CI [0.29–2.58], *p* = 0.014). Further, experiencing higher levels of emotional distress was associated with worse body image (females: *β* 0.27, 95% CI [0.21–0.33], *p* < 0.001, males: *β* 0.36, 95% CI [0.29–0.43], *p* < 0.001). Among females, baseline lower levels of education (*p* = 0.002), not currently working or studying (*p* = 0.009), and being born in Sweden (*p* = 0.005) were further found to be associated with significantly higher BIS scores.

## 4 Discussion

This study sought to investigate body image following a cancer diagnosis in young adulthood, in terms of prevalence of body image disturbance, how perceptions of body image evolve from 1.5 to 5 years following diagnosis, and factors associated with worse body image over time. More than half (54%) of females and roughly a fourth (24%) of males reported clinically significant levels of body image disturbance at 1.5 years, followed by approximately 40% and 15% at follow‐ups. Effect of time on body image differed by type of cancer in the female sample, with participants diagnosed with breast cancer and lymphoma experiencing improvement over time. Significant improvements were further observed across the entire male sample. Factors found to be associated with higher levels of body image concerns for the whole sample included intensive treatment and emotional distress.

The observed prevalence rates are in line with previous findings on body image disturbance among individuals with breast cancer [29], testicular cancer [30], brain tumors [31], and gynecological cancer [32]. While few studies have investigated body image following lymphoma, Kang et al. [33] found that 40% of adult lymphoma survivors reported dissatisfaction with their body image at a median of 2 years post hematopoietic stem cell transplantation, similar to proportions reported among males diagnosed with lymphoma at 1.5 years in the present study. Although body image concerns among male survivors have generally received limited attention, existing research suggests less impact on body image than among females [11]. This pattern was also observed in the present study, with a lower proportion of BIS cases among males, though this was not formally tested. Such differences may reflect gender norms and sociocultural expectations surrounding body image, which may further contribute to body image concerns being overlooked among males [4, 34]. Males also tend to report less impact on sexuality [35] and lower emotional distress following cancer [36], factors linked to body image in previous work [10, 11, 33]. The predominance of testicular cancer in our sample, typically treated with shorter and less intensive regimens, may further contribute to a lower overall prevalence of body image disturbance. However, prior research suggests that body image concerns may be minimized or less frequently reported among males [34], raising the possibility that such issues are underrecognized or underreported in this group [10].

Overall, previous studies indicate that body image concerns following cancer is often heightened during and shortly after treatment and gradually improves with time [12, 13]. While the present study did not capture the initial treatment and early post‐treatment period, the most pronounced changes in body image observed occurred between 1.5 and 3 years. This suggests that most adjustment to the post‐treatment body occur during the first 3 years following diagnosis, potentially reflecting cessation of temporary side‐effects from treatment and development of coping strategies [37]. However, a substantial proportion of participants in the present study continued to report BIS scores reaching the ≥ 10 the cut‐off at each assessment, highlighting the fact that for many, concerns about the body may persist long into survivorship.

Among females, body image trajectories differed by cancer type. Participants with breast cancer and lymphoma, who reported high levels of body image disturbance at 1.5 years, showed significant improvement over time. Importantly, these improvements occurred alongside a persistent burden of clinically significant body image disturbance. The relatively stable body image perceptions observed for those diagnosed with cervical, ovarian cancer or brain tumor may reflect lower initial levels of body image concerns, leaving less room for improvement, but could also indicate different underlying challenges, such as persistent symptoms or functional impairments that do not change substantially during follow‐up. The impact of breast cancer on body image is well‐documented and may reflect the invasiveness and duration of treatments, including surgery, chemo‐ and radiotherapy, and long‐term hormonal therapy [6, 38]. Approximately 50% of participants with breast cancer in the present study reported being on treatment at each assessment, with a majority receiving hormonal therapy (Table S6). Long‐term physical changes of hormonal therapy such as premature menopause and related symptoms may have implications for feminine identity, fertility and sexuality [38], and have negative impact on body image [11]. While body image among individuals with lymphoma has been less studied, findings from studies across different cancer types suggest that more intensive treatments are linked to negative impact on body image [33, 39]. In our sample, a large proportion of participants with breast cancer (∼70%) and lymphoma (∼60%) received higher levels of treatment intensity (Level 3–4 of the ITR‐YA [25]). In contrast, approximately 80% of those with cervical, ovarian, or brain tumors received less intensive treatment (Table S6). Our results were in line with previous research linking more intensive treatment [12, 39] and advanced disease [11] to poorer body image outcomes. However, differences between diagnostic groups remained when treatment intensity was included in the models, suggesting that treatment intensity does not fully explain the observed variation.

In this study, higher concurrent emotional distress was associated with worse body image among both females and males, consistent with prior research linking body image concerns to symptoms of depression and anxiety, and diminished psychological well‐being [10, 15]. This association is further particularly relevant as a majority of young adults report clinical levels of anxiety and a third meet clinical levels of depression during the first 5 years following a cancer diagnosis [36]. In the present study, the HADS summary score was used in order to maintain a parsimonious model, and symptoms of anxiety and depression were therefore not examined separately. Future research should investigate whether these symptoms contribute through distinct pathways to body image concerns following cancer. Among females, lower educational attainment and not working or studying at the first assessment, 1.5 years post‐diagnosis, was also linked to worse body image, aligning with previous, though somewhat mixed, findings. Studies have reported associations between lower educational level and worse body image outcomes [10, 39]. Employment has further been found to be associated with better health‐related quality of life (HRQoL) following cancer [40]. Given the close relationship between HRQoL and body image [6, 10, 14, 33], it is plausible that employment or educational attainment provides social interaction and sense of purpose, which may buffer against body image concerns [11].

Notably, fixed effects explained 36.6% and 27.4% of the variance in body image among females and males, respectively, while the full models, including random intercept for participants, explained over 70%. Fixed effects capture the average influence of covariates across all participants, whereas random effects capture individual‐level variability in baseline BIS scores. The substantial increase in explained variance when including random effects underscores the importance of individual differences and underscores the need for tailored psychosocial support that reflects the diverse experiences of young adults following cancer.

### 4.1 Clinical and Research Implications

A substantial proportion of young adults in this study reported body image disturbance, underscoring the need for support in managing the physical and psychological impact of cancer and its treatment. Among groups experiencing significant improvements in body image, this change was most evident between 1.5 and 3 years post‐diagnosis, suggesting that this period may be a critical window for intervention to enhance and support these improvements. At the same time, the persistence of body image disturbance reported over time highlights the need for continued assessment and support. While body image concerns should be addressed with all patients, those undergoing intensive treatment or experiencing high concurrent emotional distress may require particular attention.

Although existing psychological interventions have shown promise, especially among adult breast cancer survivors [41], few have been tailored to the unique needs of young adults or those with diverse cancer types. Given the developmental significance of body image in young adulthood and the unmet needs reported in this area [10], future research should utilize qualitative methods in exploring the needs and experiences of body image among young adults following cancer; particularly underrepresented groups such as males, and individuals with lymphoma. Such insights could inform the development and implementation of targeted interventions to reduce body image disturbance and ultimately enhance quality of survivorship. Furthermore, in light of the rising incidence of early‐onset colorectal cancer [42], further research is needed to better understand body image concerns in this emerging survivor population.

### 4.2 Study Limitations

Some limitations of the present study should be noted. First, as indicated by previous studies, treatment initiation and the time during and immediately following treatment are crucial periods with regards to body image. The first assessment of the present study occurred at 1.5 years post‐diagnosis, and does thus not capture body image at time of diagnosis and initial treatment. As such, the full trajectory of possible change in body image following diagnosis and treatment is not captured in the present study. Further, we did not assess whether participants experienced body image concerns prior to their cancer diagnosis. Since body image concerns is a common problem among young adults in general [4], controlling for pre‐cancer body image concerns could have provided a more complete picture. Second, while BIS is a well‐established instrument, it mainly covers body image concerns related to physical appearance, meaning that the full range of concerns may not have been captured. This might be particularly problematic for cancer types and treatments that do not necessarily result in primarily appearance related changes, and body image problems among such groups, including participants with lymphoma or brain tumor, may thus be underestimated. Additionally, as BIS has primarily been evaluated in breast cancer populations [22], it may be less sensitive to aspects of body image that could be more salient for males, potentially leading to underestimation in this group. Future studies should consider the selection and development of instruments that more comprehensively capture the multidimensional nature of body image in young adult cancer populations [43], including both appearance and function‐related concerns. Finally, some variables were unevenly distributed across levels, which could reduce the precision of estimates for some groups, particularly those with smaller sample sizes (e.g., ovarian cancer).

## 5 Conclusion

Body image was found to improve over time among male survivors and females diagnosed with breast cancer and lymphoma, and remain relatively stable among others. However, a substantial proportion of participants reported persistent body image disturbance between 1.5 and 5 years following a cancer diagnosis. Future research should qualitatively explore the needs and experiences of body image following cancer, particularly among previously understudied groups, in order to enable the development of timely support for alleviating problems with body image.

## Author Contributions

**Rebecca Skog:** conceptualization, methodology, formal analysis, investigation, writing – original draft, visualization. **Sarah Marklund:** conceptualization, methodology, writing – review and editing. **Claudia Lampic:** conceptualization, methodology, investigation, resources, writing – review and editing, visualization, supervision, project administration, funding acquisition. **Lena Wettergren:** conceptualization, methodology, investigation, resources, writing – review and editing, visualization, supervision, project administration, funding acquisition.

## Conflicts of Interest

The authors declare no conflicts of interest.

## Supporting information


**Figure S1:** Estimated marginal means of body image over time by cancer type among females.

**Table S1:** Differences in 1.5‐year characteristics between participants responding only to the 1.5‐year assessment vs. participants who responded to the 1.5‐year assessment plus at least one more assessment (3 or 5 yrs).

**Table S2:** Full fixed and random effects table for female participants (*n* = 663^a,b^)

**Table S3:** Estimated marginal means and pairwise comparisons for females by diagnosis.

**Table S4:** Full fixed and random effects table for male participants (*n* = 308^a,b^).

**Table S5:** Estimated marginal means and pairwise comparisons for males (*n* = 308).

**Table S6:** Treatment factors by type of cancer diagnosis.

## Data Availability

The data that support the findings of this study are available from the corresponding author upon reasonable request. The data are not publicly available due to privacy or ethical restrictions.
