# Supplementary material for: Body Image Concerns and Associated Factors up to Five Years After Cancer in Young Adulthood: A Swedish Longitudinal Population‐Based Study
Source: Psychooncology. 2026 Jul 17;35(7):e70545. doi: 10.1002/pon.70545 (PMC13379270; doi:10.1002/pon.70545)
Supplement: Supplementary file 1 — Figure S1: Estimated marginal means of body image over time by cancer type among females. [file PON-35-e70545-s007.docx]

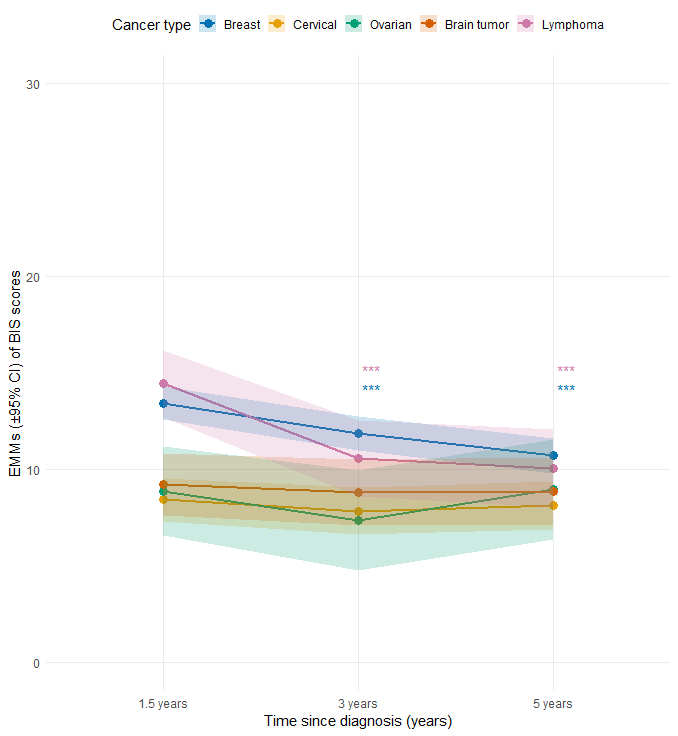


**Supplementary figure S1**. Estimated marginal means of body image over time by cancer type among females. Points represent LMM-derived estimated marginal means. **** indicates statistically significant change between 1.5 and 3 years, and 1.5 and 5 years.*
