# Supplementary material for: Body Image Concerns and Associated Factors up to Five Years After Cancer in Young Adulthood: A Swedish Longitudinal Population‐Based Study
Source: Psychooncology. 2026 Jul 17;35(7):e70545. doi: 10.1002/pon.70545 (PMC13379270; doi:10.1002/pon.70545)
Supplement: Supplementary file 2 — Table S1: Differences in 1.5‐year characteristics between participants responding only to the 1.5‐year assessment vs. participants who responded to the 1.5‐year assessment plus at least one more assessment (3 or 5 yrs). [file PON-35-e70545-s004.docx]

| **Supplementary table S1.** Differences in 1.5-year characteristics between participants responding only to the 1.5-year assessment vs. participants who responded to the 1.5-year assessment plus at least one more assessment (3 or 5 yrs) | | | | | | |
| --- | --- | --- | --- | --- | --- | --- |
|  | **Females** | | | **Males** | | |
|  | Responders 1.5 yrs  n = 146 | Responders ≥2 assessments  n = 548 | *p* | Responders 1.5 yrs  n = 77 | Responders ≥2 assessments  n = 239 | *p* |
| *Sociodemographic variables* | | |  |  |  |  |
| **Age at diagnosis, years**  Mean (SD) | 33.3 (4.9) | 33.1 (4.9) | 0.626 | 31.1 (5.0) | 30.6 (5.6) | 0.589 |
| **Birth country** |  |  | **<.001** |  |  | **0.022** |
| Sweden | 105 (71.9) | 474 (86.5) |  | 60 (77.9) | 212 (88.7) |  |
| Other country | 40 (27.4) | 74 (13.5) |  | 17 (22.1) | 26 (10.9) |  |
| **Sexual orientation** |  |  | 0.495 |  |  | 0.748 |
| Heterosexual | 133 (91.1) | 500 (91.2) |  | 70 (90.9) | 227 (95.0) |  |
| Other | 7 (4.8) | 38 (6.9) |  | 4 (5.2) | 10 (4.2) |  |
| **Partner** |  |  | 0.160 |  |  | 0.706 |
| Yes | 116 (79.5) | 469 (85.6) |  | 58 (75.3) | 187 (78.2) |  |
| No | 28 (19.2) | 78 (14.2) |  | 19 (24.7) | 52 (21.8) |  |
| **Education level** |  |  | **<.001** |  |  | 0.772 |
| Not university | 84 (57.5) | 191 (34.9) |  | 44 (57.1) | 130 (54.4) |  |
| University | 61 (41.8) | 356 (65.0) |  | 33 (42.9) | 109 (45.6) |  |
| **Occupation status** |  |  | **0.006** |  |  | **0.006** |
| Working, studying | 98 (67.1) | 432 (78.8) |  | 63 (81.8) | 206 (86.2) |  |
| Other | 47 (32.2) | 115 (21.0) |  | 14 (18.2) | 33 (13.8) |  |
| *Clinical variables* |  |  |  |  |  |  |
| **Diagnosis** |  |  | 0.308 |  |  | 0.652 |
| Breast cancer | 66 (45.2) | 283 (51.6) |  | - | - |  |
| Cervical cancer | 48 (32.9) | 142 (25.9) |  | - | - |  |
| Ovarian cancer | 6 (4.1) | 26 (4.7) |  | - | - |  |
| Brain tumor | 11 (7.5) | 55 (10.0) |  | 14 (18.2) | 43 (18.0) |  |
| Lymphoma | 15 (10.3) | 42 (7.7) |  | 17 (22.1) | 42 (17.6) |  |
| Testicular cancer | - | - |  | 46 (59.7) | 154 (64.4) |  |
| **Treatment intensity** |  |  | 0.160 |  |  | 0.148 |
| Least/moderately | 57 (39.0) | 257 (46.9) |  | 40 (51.9) | 146 (61.1) |  |
| Very/most | 82 (56.2) | 277 (50.5) |  | 37 (48.1) | 89 (37.2) |  |
| **Ongoing treatment** |  |  | 0.145 |  |  | 0.352 |
| Yes | 44 (67.8) | 206 (37.6) |  | 12 (15.6) | 26 (10.9) |  |
| No | 44 (30.1) | 339 (61.9) |  | 64 (83.1) | 212 (88.7) |  |
| *Psychological variables* |  |  |  |  |  |  |
| **HADS**  Mean (SD) | 15.2 (8.4) | 13.3 (7.1) | **0.013** | 10.9 (7.1) | 10.3 (6.9) | 0.501 |
| **Body image**  Mean (SD) | 13.8 (8.7) | 11.5 (7.9) | **0.003** | 7.0 (6.2) | 5.8 (5.9) | 0.077 |
| Differences assessed using chi-square tests/fishers exact test and two-tailed independent *t*-tests or Mann-Whitney U-tests t-tests.  Numbers do not sum up due to missing data. | | | | | | |
