# Supplementary material for: Body Image Concerns and Associated Factors up to Five Years After Cancer in Young Adulthood: A Swedish Longitudinal Population‐Based Study
Source: Psychooncology. 2026 Jul 17;35(7):e70545. doi: 10.1002/pon.70545 (PMC13379270; doi:10.1002/pon.70545)
Supplement: Supplementary file 3 — Table S2: Full fixed and random effects table for female participants (n = 663a,b) [file PON-35-e70545-s001.docx]

| **Supplementary table S2.** Full fixed and random effects table for female participants (n=663^a.b^) | | | | | | | |
| --- | --- | --- | --- | --- | --- | --- | --- |
| **Fixed effect** | **Estimate (β)** | **SE** | | **95% CI** | | ***t*** | ***p*** |
| **Time** |  |  | |  | |  |  |
| Intercept ^c^ | 13.52 | 0.82 | | 11.91 – 15.13 | | 16.52 | **<.001** |
| 3 years | -1.58 | 0.36 | | -2.28 – -0.88 | | -4.43 | **<.001** |
| 5 years | -2.73 | 0.37 | | -3.46 – -1.99 | | -7.31 | **<.001** |
| **Diagnosis** |  |  | |  | |  |  |
| Breast cancer (ref) |  |  | |  | |  |  |
| Cervical cancer | -5.02 | 0.59 | | -6.18 – -3.86 | | -8.51 | **<.001** |
| Ovarian cancer | -4.56 | 1.23 | | -7.05 – -2.07 | | -3.70 | **<.001** |
| Brain tumor | -4.22 | 0.89 | | -6.00 – -2.45 | | -4.71 | **<.001** |
| Lymphoma | 0.99 | 0.99 | | -0.99 – 2.97 | | 1.00 | 0.320 |
| **Birth country** |  |  | |  | |  |  |
| Sweden (ref) |  |  | |  | |  |  |
| Other country | -1.67 | 0.59 | | 0.50 – 2.83 | | 2.83 | **0.005** |
| **Emotional distress – deviation** ^d^ | 0.27 | 0.03 | | 0.21 – 0.33 | | 8.65 | **<.001** |
| **Emotional distress 1.5 years ^d^** | 0.50 | 0.03 | | 0.44 – 0.57 | | 15.95 | **<.001** |
| **Education 1.5 years**  Not university (ref)  University | -1.44 | 0.47 | | -2.37 – -0.52 | | -3.08 | **0.002** |
| **Occupation 1.5 years** |  |  | |  | |  |  |
| Not working/studying (ref)  Working/studying | -1.42 | 0.54 | | -2.48 – -0.35 | | -2.62 | **0.009** |
| **Intensity of treatment** |  |  | |  | |  |  |
| Least/moderately (ref)  Very/most | 1.57 | 0.48 | | 0.63 – 2.51 | | 3.29 | **0.001** |
| **Time x diagnosis interaction term ^e^** | | | | | | | |
| Cervical cancer | | | | | | | |
| 3 years | 0.99 | 0.55 | | -0.10 – 2.09 | | 1.79 | 0.075 |
| 5 years | 2.43 | 0.56 | | 1.31 – 3.54 | | 4.29 | **<.001** |
| Ovarian cancer | | | | | | | |
| 3 years | 0.05 | 1.35 | | -2.72 – 2.83 | | 0.04 | 0.968 |
| 5 years | 2.82 | 1.39 | | -0.03 – 5.68 | | 2.04 | 0.052 |
| Brain tumor | | | | | | | |
| 3 years | 1.17 | 0.89 | | -0.60 – 2.94 | | 1.32 | 0.192 |
| 5 years | 2.37 | 1.03 | | 0.31 – 4.43 | | 2.30 | **0.024** |
| Lymphoma | | | | | | | |
| 3 years | -2.28 | 1.04 | | -4.38 – -0.19 | | -2.18 | **0.033** |
| 5 years | -1.65 | 1.16 | | -3.98 – 0.67 | | -1.43 | 0.159 |
| **Random effects** |  |  | |  | |  |  |
| **Grouping factor** | **Effects** | | **Variance** | | | **SD** | |
| Id | Intercept | | 22.75 | | | 4.78 | |
| Residual |  | | 15.31 | | | 3.91 | |
| **Model fit** |  | |  | | |  | |
| R2 | **Marginal** | | | | **Conditional** | | |
|  | 0.366 | | | | 0.745 | | |
| ^a^ Total number of observations: 1589  ^b^ 31 participants were excluded due to missing either all outcome data or covariates  ^c^ The intercept represents the expected BIS score at the reference level/zero of all variables  ^d^ HADS was separated into (i) a variable representing scores at 1.5 years (included to account for differences between responders 1.5 yrs and responders >2 assessments) and (ii) a variable representing within-person deviation from the 1.5-year score.  ^e^ For time x diagnosis interaction, the reference group and time point is breast cancer at 1.5 years | | | | | | | |
