# Supplementary material for: Body Image Concerns and Associated Factors up to Five Years After Cancer in Young Adulthood: A Swedish Longitudinal Population‐Based Study
Source: Psychooncology. 2026 Jul 17;35(7):e70545. doi: 10.1002/pon.70545 (PMC13379270; doi:10.1002/pon.70545)
Supplement: Supplementary file 4 — Table S3: Estimated marginal means and pairwise comparisons for females by diagnosis. [file PON-35-e70545-s003.docx]

| **Supplementary table S3.** Estimated marginal means and pairwise comparisons for females by diagnosis | | | | | | | | | | |
| --- | --- | --- | --- | --- | --- | --- | --- | --- | --- | --- |
|  | **Adjusted means** | | | | **Pairwise comparisons** | | | | | |
| **Diagnosis** | **Time** | **Adjusted mean** | **SE** | **95% CI** | **Comparison** | **Mean difference** | **SE** | **95% CI** | **p (adj.)** | **Cohen’s d** |
| **Breast cancer** | 1.5 years | 13.45 | 0.44 | 12.58 – 14.31 | 1.5 – 3 years | 1.58 | 0.34 | 0.92 – 2.24 | **<.001** | 0.40 |
|  | 3 years | 11.87 | 0.47 | 10.94 – 12.80 | 3 – 5 years | 1.15 | 0.36 | 0.44 – 1.86 | **0.004** | 0.29 |
|  | 5 years | 10.72 | 0.46 | 9.82 – 11.61 | 1.5 – 5 years | 2.73 | 0.35 | 2.04 – 3.41 | **<.001** | 0.70 |
| **Cervical cancer** | 1.5 years | 8.43 | 0.50 | 7.44 – 9.42 | 1.5 – 3 years | 0.59 | 0.49 | -0.38 – 1.56 | 0.463 | 0.15 |
|  | 3 years | 7.84 | 0.56 | 6.74 – 8.94 | 3 – 5 years | -0.28 | 0.54 | -1.34 – 0.78 | 0.859 | -0.07 |
|  | 5 years | 8.12 | 0.54 | 7.06 – 9.19 | 1.5 – 5 years | 0.30 | 0.51 | -0.69 – 1.30 | 0.822 | 0.08 |
| **Ovarian cancer** | 1.5 years | 8.89 | 1.17 | 6.60 – 11.18 | 1.5 – 3 years | 1.52 | 1.19 | -0.81 – 3.86 | 0.406 | 0.39 |
|  | 3 years | 7.36 | 0.94 | 5.52 – 9.20 | 3 – 5 years | -0.28 | 1.26 | -4.10 – 0.85 | 0.404 | -0.41 |
|  | 5 years | 8.98 | 1.12 | 6.79 – 11.18 | 1.5 – 5 years | 0.30 | 1.19 | -2.43 – 2.24 | 0.996 | -0.02 |
| **Brain tumor** | 1.5 years | 9.22 | 0.80 | 7.65 – 10.80 | 1.5 – 3 years | 0.41 | 0.75 | -1.07 – 1.89 | 0.849 | 0.10 |
|  | 3 years | 8.81 | 0.84 | 7.17 – 10.46 | 3 – 5 years | -0.06 | 0.81 | -1.65 – 1.53 | 0.997 | -0.01 |
|  | 5 years | 8.87 | 0.93 | 7.05 – 10.69 | 1.5 – 5 years | 0.35 | 0.79 | -1.20 – 1.91 | 0.896 | 0.09 |
| **Lymphoma** | 1.5 years | 14.44 | 0.95 | 12.58 – 16.30 | 1.5 – 3 years | 3.86 | 0.88 | 2.13 – 5.59 | **<.001** | 0.99 |
|  | 3 years | 10.58 | 1.07 | 8.48 – 12.68 | 3 – 5 years | 0.52 | 0.96 | -1.37 – 2.41 | 0.850 | 0.13 |
|  | 5 years | 10.06 | 1.04 | 8.02 – 12.10 | 1.5 – 5 years | 4.38 | 0.89 | 2.64 – 6.13 | **<.001** | 1.12 |
| Pairwise comparisons adjusted for multiple comparisons using Tukey method.  Cohen’s d was calculated from model-based estimated marginal means with pooled SD | | | | | | | | | | |
