# Supplementary material for: Body Image Concerns and Associated Factors up to Five Years After Cancer in Young Adulthood: A Swedish Longitudinal Population‐Based Study
Source: Psychooncology. 2026 Jul 17;35(7):e70545. doi: 10.1002/pon.70545 (PMC13379270; doi:10.1002/pon.70545)
Supplement: Supplementary file 5 — Table S4: Full fixed and random effects table for male participants (n = 308a,b). [file PON-35-e70545-s002.docx]

| **Supplementary table S4.** Full fixed and random effects table for male participants (n=308^a,b^) | | | | | | | |
| --- | --- | --- | --- | --- | --- | --- | --- |
| **Fixed effect** | **Estimate (β)** | **SE** | | **95% CI** | | ***t*** | ***p*** |
| **Time** |  |  | |  | |  |  |
| Intercept ^c^ | 4.60 | 0.82 | | 2.96 – 6.25 | | 5.61 | **<.001** |
| 3 years | -0.87 | 0.27 | | -1.39 – -0.34 | | -3.27 | **0.001** |
| 5 years | -1.06 | 0.30 | | -1.65 – -0.47 | | -3.55 | **<.001** |
| **Diagnosis** |  |  | |  | |  |  |
| Testicular cancer (ref) |  |  | |  | |  |  |
| Brain tumor | 0.74 | 0.67 | | -0.58 – 2.07 | | 1.12 | 0.268 |
| Lymphoma | 0.97 | 0.80 | | -0.62 – 2-56 | | 1.21 | 0.229 |
| **Birth country** |  |  | |  | |  |  |
| Sweden (ref) |  |  | |  | |  |  |
| Other | 0.45 | 0.73 | | -1.02 – 1.92 | | 0.62 | 0.539 |
| **Age at diagnosis** | -0.07 | 0.04 | | -0.15 – 0.02 | | -1.54 | 0.125 |
| **Emotional distress** | 0.36 | 0.03 | | 0.29 – 0.43 | | 10.34 | **<.001** |
| **Occupation 1.5 years** |  |  | |  | |  |  |
| Not working/studying (ref)  Working/studying | 0.29 | 0.80 | | -1.30 – 1.88 | | 0.37 | 0.716 |
| **Intensity of treatment** |  |  | |  | |  |  |
| Least/moderately (ref)  Very/most | 1.44 | 0.58 | | 0.29 – 2.58 | | 2.48 | **0.014** |
| **Ongoing treatment** | | | | | | | |
| No (ref) |  |  | |  | |  |  |
| Yes | 1.15 | 0.75 | | -0.36 – 2.66 | | 1.54 | 0.131 |
| **Random effects** |  |  | |  | |  |  |
| **Grouping factor** | **Effects** | | **Variance** | | | **SD** | |
| Id | Intercept | | 13.45 | | | 3.67 | |
| Residual |  | | 8.10 | | | 2.85 | |
| **Model fit** |  | |  | | |  | |
| R2 | **Marginal** | | | | **Conditional** | | |
|  | 0.274 | | | | 0.727 | | |
| ^a^ Total number of observations: 702  ^b^ 8 participants were excluded due to missing either all outcome data or covariates  ^c^ The intercept represents the expected BIS score at the reference level/zero of all variables. | | | | | | | |
