# Supplementary material for: Body Image Concerns and Associated Factors up to Five Years After Cancer in Young Adulthood: A Swedish Longitudinal Population‐Based Study
Source: Psychooncology. 2026 Jul 17;35(7):e70545. doi: 10.1002/pon.70545 (PMC13379270; doi:10.1002/pon.70545)
Supplement: Supplementary file 6 — Table S5: Estimated marginal means and pairwise comparisons for males (n = 308). [file PON-35-e70545-s005.docx]

| **Supplementary table S5.** Estimated marginal means and pairwise comparisons for males (n=308) | | | | | | | | | |
| --- | --- | --- | --- | --- | --- | --- | --- | --- | --- |
| **Estimated marginal means** | | | | **Pairwise comparisons** | | | | | |
| **Time** | **Adjusted mean** | **SE** | **95% CI** | **Comparison** | **Mean difference** | **SE** | **95% CI** | **p (adj.)** | **Cohen’s d** |
| 1.5 years | 6.85 | 0.47 | 5.92 – 7.78 | 1.5 – 3 years | 0.87 | 0.27 | 0.33 – 1.40 | **0.004** | 0.30 |
| 3 years | 5.98 | 0.51 | 4.98 – 6.98 | 3 – 5 years | 0.19 | 0.30 | -0.39 – 0.78 | 0.789 | 0.07 |
| 5 years | 5.79 | 0.52 | 4.76 – 6.81 | 1.5 – 5 years | 1.06 | 0.28 | 0.51 – 1.62 | **<.001** | 0.37 |
| Pairwise comparisons adjusted for multiple comparisons using Tukey method.  Cohen’s d was calculated from model-based estimates. | | | | | | | | | |
