# Supplementary material for: Body Image Concerns and Associated Factors up to Five Years After Cancer in Young Adulthood: A Swedish Longitudinal Population‐Based Study
Source: Psychooncology. 2026 Jul 17;35(7):e70545. doi: 10.1002/pon.70545 (PMC13379270; doi:10.1002/pon.70545)
Supplement: Supplementary file 7 — Table S6: Treatment factors by type of cancer diagnosis. [file PON-35-e70545-s006.docx]

| **Supplementary table S6.** Treatment factors by type of cancer diagnosis | | | | | | | | |
| --- | --- | --- | --- | --- | --- | --- | --- | --- |
|  | **Breast cancer** | **Cervical cancer** | **Ovarian cancer** | **Brain tumor** | | **Lymphoma** | | **Testicular cancer** |
|  |  |  |  | **Females** | **Males** | **Females** | **Males** |  |
|  | n (%) | n (%) | n (%) | n (%) | n (%) | n (%) | n (%) | n (%) |
| **Ongoing cancer treatment** | |  |  |  |  |  |  |  |
| **1.5 years** |  |  |  |  |  |  |  |  |
| Yes ^a^ | 232 (67.0) ^b^ | 8 (4.3) | 2 (6.2) | 5 (7.6) | 20 (35.1) | 3 (5.3) | 9 (15.2) | 9 (4.5) |
| **3 years** |  |  |  |  |  |  |  |  |
| Yes ^a^ | 147 (57.0) ^c^ | 1 (0.8) | 1 (4.3) | 5 (9.2) | 2 (5.3) | 2 (5.4) | 2 (5.3) | 4 (2.9) |
| **5 years** |  |  |  |  |  |  |  |  |
| Yes ^a^ | 118 (49.6) ^d^ | 1 (0.8) | 1 (4.5) | 1 (2.2) | 1 (2.9) | 2 (5.9) | 4 (11.8) | 4 (3.2) |
| **Treatment intensity ^e^** |  |  |  |  |  |  |  |  |
| **1.5 years** |  |  |  |  |  |  |  |  |
| Least/moderately | 84 (24.3) | 135 (77.6) | 26 (83.9) | 49 (75.4) | 36 (64.3) | 20 (35.1) | 9 (15.5) | 141 (71.2) |
| Very/most | 262 (75.7) | 39 (22.4) | 5 (16.1) | 16 (24.6) | 20 (35.7) | 37 (64.9) | 49 (84.5) | 57 (28.8) |
| **3 years** |  |  |  |  |  |  |  |  |
| Least/moderately | 68 (26.4) | 93 (79.0) | 20 (91.3) | 43 (81.1) | 25 (67.6) | 14 (36.8) | 6 (15.8) | 98 (71.0) |
| Very/most | 189 (73.6) | 25 (21.0) | 2 (8.7) | 10 (18.9) | 12 (32.4) | 23 (63.2) | 32 (84.2) | 40 (29.0) |
| **5 years** |  |  |  |  |  |  |  |  |
| Least/moderately | 61 (25.5) | 87 (78.4) | 18 (85.7) | 36 (80.0) | 26 (72.2) | 13 (37.1) | 6 (18.2) | 88 (71.5) |
| Very/most | 178 (74.5) | 24 (21.6) | 3 (14.3) | 9 (20.0) | 10 (27.8) | 22 (62.9) | 27 (81.8) | 35 (28.5) |
| ^a^ Includes all form of ongoing treatment (chemotherapy, radiotherapy, hormonal therapy, other)  ^b^ 58.1% of those on treatment reported currently being on hormonal therapy  ^c^ 52.3% of those on treatment reported currently being on hormonal therapy  ^d^ 49.6% of those on treatment reported currently being on hormonal therapy  ^e^ Classified according to the Intensity of Treatment Rating Scale (ITR-YA) | | | | | | | | |
